# Comparison of Two Protocols of Carbon Tetrachloride-Induced Cirrhosis in Rats – Improving Yield and Reproducibility

**DOI:** 10.1038/s41598-018-27427-9

**Published:** 2018-06-15

**Authors:** José I Fortea, Carolina Fernández-Mena, Marta Puerto, Cristina Ripoll, Jorge Almagro, Juan Bañares, José M. Bellón, Rafael Bañares, Javier Vaquero

**Affiliations:** 1 Servicio de Aparato Digestivo, Hospital General Universitario Gregorio Marañón, Madrid, Spain; 2 Instituto de Investigación Sanitaria Gregorio Marañón (IiSGM), Madrid, Spain; 3 Centro de Investigación Biomédica en Red de Enfermedades Hepáticas y Digestivas (CIBEREHD), Madrid, Spain; 4 Innere Medizin I, Martin-Luther-Universität Halle-Wittenberg, Halle, Germany; 5 Department of Statistics, IiSGM, Madrid, Spain; 6 Facultad de Medicina, Universidad Complutense, Madrid, Spain

## Abstract

Despite being a cardinal experimental model, the induction of cirrhosis in rats by repeated exposure to carbon tetrachloride (CCl4) has low reproducibility. Here, we compared two models of cirrhosis induced by orogastric administration of CCl4 once (CCl4-1xWk) or twice a week (CCl4-2xWk) for 12 weeks in male Sprague-Dawley rats. Control rats received water instead of CCl4. Both CCl4 protocols similarly attenuated body weight gain (p < 0.01 vs. Control). Although both CCl4 protocols increased hepatic fibrosis, portal hypertension and splenomegaly, the magnitude of these alterations was higher and more consistent in CCl4-2xWk rats. Importantly, two CCl4-1xWk rats did not develop cirrhosis versus a 100% yield of cirrhosis in CCl4-2xWk rats. The CCl4-2xWk protocol consistently induced liver atrophy together with hematological, biochemical and coagulation abnormalities characteristic of advanced cirrhosis that were absent in CCl4-1xWk rats. Ascites occurred in 20% and 80% of rats in theCCl4-1xWk and CCl4-2xWk groups (p < 0.01). All rats showed normal renal function, arterial blood gases and stable systemic hemodynamics. The total dose of CCl4 and mortality rate were similar in both protocols. The CCl4-2xWk protocol, therefore, was highly reproducible and effective for the induction of experimental cirrhosis within a confined time, representing a valuable advance for liver research.

## Introduction

Experimental animal models are key for our understanding of the mechanisms responsible of hepatic fibrogenesis, portal hypertension and their complications^1^. Examples of pioneering revelations from these models include the primary role of portal hyperemia in the pathogenesis of portal hypertension^2^ or the dynamic nature of hepatic fibrogenesis^3^. Among the animal models of hepatic fibrosis and cirrhosis, the most widely used is the rat with repeated exposition to carbon tetrachloride (CCl4), which closely resembles the histological and hemodynamic features of human disease^4,5^.

Acute CCl4 administration causes consistent hepatotoxicity due to reactive metabolites generated by cytochrome P-450 enzymes, primarily CYP2E1, expressed in perivenular hepatocytes. With repeated administration, the recurring episodes of acute injury lead to centrilobular necrosis accompanied by inflammation and hepatic stellate cell activation, enhancing the synthesis of extracellular matrix and resulting in the architectural alterations that define cirrhosis^4,5^. Portal hypertension and hepatic decompensation also develop when CCl4 administration is sufficiently prolonged^6^. Although several routes can be used for administering CCl4, the orogastric route is generally preferred because of its low equipment requirements, safety, inexpensiveness, and avoidance of intra-abdominal adhesions. Despite the diverse delivery options and its widespread use, the reproducibility and associated mortality of CCl4 administration for induction of cirrhosis remain unsatisfactory.

Major problems of the experimental models of CCl4-induced cirrhosis are the unpredictable acute damage caused by CCl4 in each rat (particularly in initial doses), and the highly variable yield of cirrhosis^7^. Attempts to overcome these pitfalls include the use of cytochrome P450 inducers (e.g. phenobarbital in the drinking water)^8^, the individualization of CCl4 doses according to the body weight or to the changes in body weight of the animal^7,9^, and diverse duration and schedules of CCl4 administration such as once^6,7^, twice^10^ or thrice^11,12^ a week or others^9,13^. Such variety of protocols reflect that the prior problems are still present. One of the most commonly used protocols is the one reported by Runyon *et al*.^6^, which is based on the administration of CCl4 by oral gavage once a week at a dose adjusted to the change of body weight 48 h after the last dose. Although high yields of cirrhosis (100%) and ascites (>90%) have been reported using this protocol, the associated mortality is consistently high (40–60%) and the duration of CCl4 administration required to develop ascites is extremely variable (6 to 20 weeks) from one rat to another even within single studies^6,14–18^. In 2008, Regimbeau *et al*. reported a “rapid” protocol for the induction of cirrhosis involving the administration of CCl4 by oral gavage twice a week, with the CCl4 dose being adjusted to the body weight on the same day of treatment^9^. Although the reported yield of cirrhosis was 100% with an associated mortality of 30%, the later protocol has only been scarcely used by researchers. Importantly, there are no studies comparing two different protocols of CCl4-induced cirrhosis in parallel.

Here, we performed a comprehensive comparison between Runyon’s protocol (CCl4-1xWk)^6^ and a modified version of the “rapid” protocol reported by Regimbeau *et al*. (CCl4-2xWk)^9^. Importantly, the CCl4-2xWk protocol showed major improvements in terms of reproducibility and yield of cirrhosis, portal hypertension and ascites without increasing mortality.

## Material and Methods

### Animals

Sprague-Dawley rats (*Rattus norvegicus*, Charles River Laboratories) were bred in our animal facilities. Male experimental (F1) animals (130–200 grams body weight, 5–6 week-old) were maintained under constant conditions of temperature, air humidity, and a 12:12-hour light:dark schedule. All rats were allowed 7–10 days of acclimation to the room and to manipulation prior to the start of oral gavaging, and they had free access to standard chow and tap water throughout the experiments. All studies were approved by the Ethics Committee for Animal Experimentation of Hospital General Universitario Gregorio Marañón, and were conducted in conformity with the European Union Directive 2010/63/EU and the RD53/2013 of Ministerio de la Presidencia of Spain.

### Experimental protocols for induction of cirrhosis

The rats were randomly divided in three groups: a) Rats receiving oral gavage with water (Control group, n = 11), b) Rats receiving CCl4 once a week (CCl4-1xWk group, n = 12), and c) Rats receiving CCl4 twice a week (CCl4-2xWk group, n = 15). Phenobarbital (35 gr/dl) was added to the drinking water of all rats from 2 weeks prior to the administration of CCl4/water until the termination of the experiments. CCl4 (diluted 1:1 with water, Sigma-Aldrich − 99.9% pure) or water alone were administered by orogastric intubation with a metal cannula without anesthesia or prior fasting.

Rats were treated for 12 weeks or until the development of overt ascites according to the following protocols (see Table 1):

- **Control group**: Rats received 0.5 ml of tap water once weekly (on Mondays). Body weight was measured before each administration and 4 days later (Monday and Friday).
- **CCl4-1xWk group**: Rats received CCl4 following the protocols published by Proctor and Chatamra^7^, later modified by Runyon *et al*.^6^. Briefly, the initial dose of CCl4 was 0.04 ml, and subsequent doses were administered once weekly (on Mondays) and adjusted based on the change in body weight measured 48 hours after the last dose. Each CCl4 dose was mixed with 0.5 ml of water before its administration, as opposed to the undiluted administration of CCl4 in the original protocol of Runyon *et al*. Body weight was measured before each administration and 2 and 4 days later (Monday, Wednesday and Friday).
- **CCl4-2xWk group**: Rats received CCl4 twice weekly for 12 weeks, with the CCl4 dose being adjusted on the body weight of the rats before each administration (see Table 1), based on the dosing reported in the 6-wk “rapid protocol” of Regimbeau *et al*.^9^ with some modifications. In particular, CCl4 was diluted in 0.5 ml of water, and the doses were given on Monday and Fridays (in the original Regimbeau’s protocol CCl4 was diluted 1:1 in olive oil and doses were given every 4 days).

**Table 1:** Dosage of CCl4 according to body weight in each protocol.

CCl4-1xWk PROTOCOL			CCl4-2xWk PROTOCOL	
BW change 48 h after prior dose	Dose of CCl4		BW in the day of CCl4 administration	Dose of CCl4
	<6 weeks of treatment	>6 weeks of treatment		
Stable or increasing	Increase by 0.06 ml	Increase by 0.08 ml	**150 to 230 g**	0.20 ml/kg
2–5.9% loss	Increase by 0.04 ml	Increase by 0.06 ml	**231 to 280 g**	0.25 ml/kg
6–10% loss	Increase by 0.02 ml	Increase by 0.04 ml	**281 to 310 g**	0.30 ml/kg
10.1–15% loss	Stable dose	Stable dose	**311 to 340 g**	0.35 ml/kg
>15% loss	Decrease by 0.04 ml	Decrease by 0.04 ml	**341 to 370 g**	0.40 ml/kg
			**371 to 390 g**	0.45 ml/kg

The doses of CCl4 in the CCl4-1xWk and the CCl4-2xWk protocols were based, respectively, on the studies by Runyon *et al*.^6^ and Regimbeau *et al*.^9^. In the CCl4-1xWk protocol, the initial dose of CCl4 was 0.04 ml. The volumes reflected in the table refer only to CCl4. All doses were mixed with 0.5 ml of water.

Abbreviations: BW, body weight.

### Hemodynamic measurements

A hemodynamic study was performed two weeks after the last dose of CCl4, a washout period intended to avoid the interference of inflammation associated with acute hepatic injury. The rats were anesthetized with sevoflurane (Abbott Laboratories), and the right common carotid artery and external jugular vein were dissected and canalized using a 24 G Abbocath catheter (B. Braun) and a polyethylene tube (PE50), respectively, to measure the mean arterial pressure (MAP) and the central venous pressure (CVP). Thereafter, a mid-laparotomy was performed and a 24 G Abbocath catheter (B. Braun) was inserted into the ileocolic vein to measure the portal pressure (PP). After 5 minutes of stabilization, blood pressures were registered for 5 minutes using pressure transducers and a multichannel PowerLab 8/35 and Lab Chart Reader software (AD Instruments) for analysis. Body temperature was monitored with a thermometer and maintained at stable levels with a warming pad throughout the experiment.

### Hematological, biochemical and coagulation blood tests

Immediately after the hemodynamic measurements, arterial blood was collected in tubes containing EDTA, lithium heparin or citrate. Blood cell counting, biochemical, coagulation, and arterial blood gases analyses were performed in automated analyzers. To confirm the platelet count and to assess if platelet aggregates were present, blood smears stained following the May-Grünwald method (Merck Millipore) were also evaluated.

### Histology and quantification of fibrosis

The diagnosis of cirrhosis was based exclusively in histological criteria, namely the presence of architectural distortion of the liver characterized by the formation of regenerative nodules of hepatocytes surrounded by fibrous tissue. After the hemodynamic study and the collection of blood samples, the liver and the spleen were quickly excised and weighed. Portions of the left lateral and the median lobes of the liver were placed in 10% neutral buffered formalin, and processed for paraffin embedding. Tissue sections (6 μmm thick) were cut in a microtome, and stained with Hematoxylin-Eosin, Sirius red (Direct Red 80, Sigma-Aldrich) or Masson Trichrome (Bio-Optica Milano SpA). The stained slides were assessed in a fashion blinded to the groups by trained hepatologists for establishing the diagnosis of cirrhosis (JV) and for the quantification of fibrosis (JIF). For the later, digital images were captured with a Nikon Digital Camera DXM1200F coupled to a Nikon Eclipse E800 microscope, and the area of fibrosis was quantified in thirty 20x-magnification fields (Masson trichrome stain) or fifteen 10x-magnification fields (Sirius Red stain) per specimen. An RGB (Red, Green, Blue) threshold was used to identify the areas of fibrosis using ImageJ NIH software. Fibrosis was expressed as percentage (%) of total area.

### Statistical analysis

Quantitative variables were expressed as median [interquartile range] and qualitative variables as proportions (%), unless otherwise noted. One-way ANOVA followed by Tukey’s post-hoc tests were used to assess differences between groups. In case of heterogeneity of variances, a Kruskal Wallis test followed by Dunn’s post-hoc tests were used instead. In some cases of heterogeneity of variances (e.g. Sirius and Masson), a logarithmic transformation of the variables was performed prior to analysis. Comparisons of the evolution of body weight gain were analyzed by a Mixed-Model, with group and day as fixed effects and the rat identifier as a random effect, dismissing the baseline value (zero), and including the square of the variable “day” as a covariate and the interaction day-group. All comparisons were two-tailed, and a p value < 0.05 was considered statistically significant. The analyses were performed with GraphPad Prism v7 or IBM SPSS v21.

### Data availability statement

Authors agree to make materials, data and associated protocols promptly available to readers upon requirement.

## Results

### General characteristics and mortality of the two experimental protocols of cirrhosis

**Figure 1:**
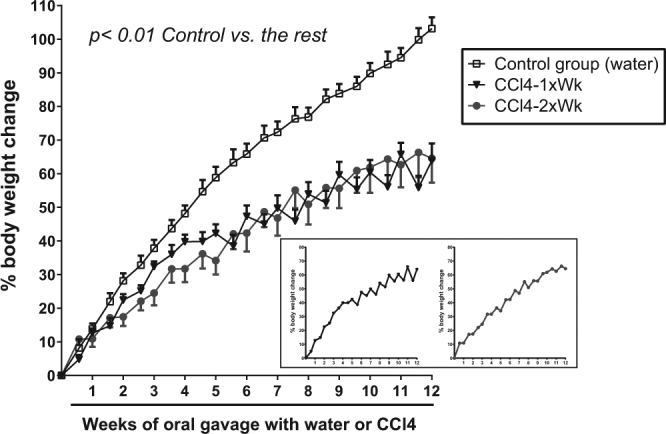
Body weight change (%) from baseline during the 12 weeks of administration of water or carbon tetrachloride (CCL4) by oral gavage. Rats received oral gavage with water (Control group) or with CCl4 once a week (CCl4-1xWk group) or twice a week (CCl4-2xWk) following the protocols detailed in the manuscript. Inset: Note the serrated shape of the curve reflecting marked losses of body weight with posterior recovery after each CCl4 dose in rats following the CCl4-1xWk protocol (left graph) versus the smoother curve in rats following the CCl4-2xWk protocol (right graph). p = 0.007 Control vs. CCl4-1xWk, p = 0.005 Control vs. CCl4-2xWk (Mixed Linear Model with Bonferroni correction for multiple comparisons).

The body weight was similar in the three groups of rats at the beginning of the administration of water or CCl4 by oral gavage (Control: 238 g [233, 250] vs. CCl4-1xWk: 259 g [243, 279] vs. CCl4-2xWk: 249 g [234, 259], p = 0.15 ANOVA). Administration of CCl4 markedly impacted on the body weight gain during the 12-week period of oral gavage. Compared with Control rats receiving water, body weight gain was markedly diminished in the two groups of rats receiving CCl4 (Mean body weight gain (mean SD [95% CI]) estimated at the 8^th^ week of follow-up: Control: 61.1 ± 4.8% [51.3, 70.9] vs. CCl4-1xWk: 41.5 ± 3.4% [34.6, 48.4] vs. CCl4-2xWk: 41.2 ± 3.1% [35.0, 47.4], p = 0.007 Control vs. CCl4-1xWk, p = 0.005 Control vs. CCl4-2xWk, Fig. 1). Consequently, body weight gain was lower at the end of the 12-week treatment period in both CCl4 groups (median [IQR]: Control: 85% [76, 115], CCl4 1xWk: 59% [15, 78], CCl4 2xWk: 48% [21, 70], p < 0.001 Control vs. CCl4 1xWk, p < 0.0001 Control vs. CCl4 2xWk). Although the impairment of body weight gain was similar in both CCl4 protocols, the rats receiving CCl4 once a week presented a highly serrated curve of body weight gain that contrasted with the smooth curve observed in the rats of the CCl4 2xWk group (Fig. 1 inset).

Although the single doses of CCl4 were lower in CCl4-2xWk rats, the total amount of CCl4 at the end of the 12-week treatment period was similar in both protocols (CCl4-1xWk: 3.6 ml [2.8, 4.0] vs. CCl4-2xWk: 3.1 ml [2.4, 3.9], p = 0.27).

Mortality was also similar in both CCl4 protocols (CCl4-1xWk: 17% vs. CCl4-2xWk: 33%, p = 0.41). None of the control rats receiving water died. In the CCl4-1xWk group, 2 rats died during the wash-out period (13^th^ week). In the CCl4-2xWk group, there were 3 deaths due to accidental instillation of CCl4 into the trachea, and 2 deaths at the 2^nd^ and 10^th^ week of induction of cirrhosis. At the end of the study, there were 11 rats in the Control group and 10 rats in each of the CCl4 groups.

### Hematological, biochemical and coagulation parameters

**Figure 2:**
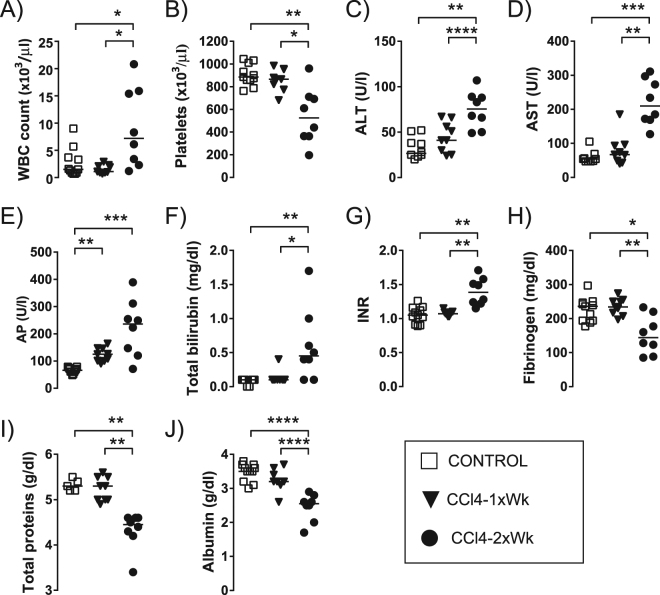
Hematological, biochemical and coagulation parameters. The panels show the (**A**) White blood cell (WBC) count, (**B**) Platelet count, (**C**) Alanine aminotransferase (ALT), (**D**) Aspartate aminotransferase (AST), (**E**) Alkaline phosphatase (AP), (**F**) Total bilirubin, (**G**) International Normalized Ratio (INR), (**H**) Fibrinogen concentration, (**I**) Total protein concentration, and (**J)** Albumin concentration in arterial blood of rats receiving oral gavage with water (Control group), CCl4 once a week (CCl4-1xWk group) or CCl4 twice a week (CCl4-2xWk) for 12 weeks following the protocols detailed in the manuscript. Horizontal lines represent the median of each group. *p < 0.05, **p < 0.01, ***p < 0.001, ****p < 0.0001 (ANOVA with Tukey’s post-hoc tests or Kruskal-Wallis with Dunn’s post-hoc tests).

Only rats receiving oral gavage with CCl4 twice a week presented blood analytical alterations suggestive of advanced liver disease. Compared with the Control and CCl4-1xWk groups, the rats in the CCl4-2xWk group showed leukocytosis (p < 0.05), thrombocytopenia (p < 0.05), biochemical alterations suggestive of liver damage (increased alanine aminotransferase and aspartate aminotransferase, both p < 0.01) and of decreased liver function, including parameters of cholestasis [increased alkaline phosphatase (p < 0.001) and bilirubin (p < 0.05)] and parameters reflecting poor synthetic function, such as increased INR (p < 0.01) and decreased circulating concentrations of fibrinogen (p < 0.05), total proteins (p < 0.01) and albumin (p < 0.001) (Fig. 2 and Table 2). Rats in the CCl4-1xWk group only showed increased levels of alkaline phosphatase compared with Control rats (p < 0.01) (Fig. 2E and Table 2). There were no significant alterations of renal function parameters, electrolytes, arterial blood gases or arterial lactate in any of the CCl4 groups compared with the Control group (Table 2).

**Table 2:** Hematological and biochemical parameters in arterial blood from rats in the Control, CCl4-1xWk and CCl4-2xWk groups.

	Control (n = 11)	CCl4-1xWk (n = 10)^#^	CCl4-2xWk (n = 10)^#^
**Blood cell count**			
*Red blood cells (x10* ^6^ */μl)*	7.7 [7.2, 8.0]	7.6 [7.5, 7.8]	6.9 [6.4, 7.5]
*Hemoglobin (g/dl)*	14.0 [13.6, 14.6]	13.6 [13.3, 14.2]	13.2 [12.5, 13.6]^*^
*Hematocrit (%)*	40.2 [38.7, 41.7]	39.8 [38.7, 40.8]	38.8 [38.1, 40.6]
*MCV (fl)*	54.3 [51.2, 54.9]	52.3 [50.6, 54.4]	56.7 [54.1, 61.0]^θ^
*White blood cells (x10* ^3^ */μl)*	1.5 [0.9, 3.7]	1.1 [0.9, 2.2]	7.2 [2.6, 15.8]^*,θ^
*Platelets (x10* ^3^ */μl)*	886 [859, 1011]	866 [798, 955]	525 [363, 706]^**,θ^
**Coagulation**			
*Prothrombin time (s)*	11.5 [10.0, 12.6]	11.7 [11.2, 11.9]	15.1 [13.3, 17.0]^**,θθ^
*INR*	1.05 [0.91, 1.16]	1.07 [1.05, 1.10]	1.39 [1.23, 1.56]^**,θθ^
*Fibrinogen (mg/dl)*	236 [194, 247]	234 [212, 253]	144 [97, 209]^*,θθ^
**Blood biochemistry**			
*Glucose (mg/dl)*	197 [166, 259]	163 [160, 201]	122 [101, 168]^**^
*ALT (U/l)*	27 [25, 41]	41 [28, 61]	76 [54, 89] ^****, θθ^
*AST (U/l)*	54 [47, 63]	67 [46, 95]	210 [170, 291]^***,θθ^
*Total Bilirubin (mg/dl)*	0.10 [0.03, 0.10]	0.10 [0.10, 0.10]	0.45 [0.18, 0.90]^**,θ^
*AP (U/l)*	66 [60, 75]	125 [101, 147]^**^	236 [127, 297]^**^
*Albumin (g/dl)*	3.5 [3.2, 3.7]	3.2 [3.2, 3.5]	2.6 [2.1, 2.8]^****,θθθθ^
*Total Proteins (g/dl)*	5.3 [5.2, 5.5]	5.3 [5.0, 5.5]	4.5 [4.2, 4.6]^**,θθ^
*LDH (U/l)*	138 [110, 214]	155 [92, 210]	207 [198, 390]^θ^
*Creatinine (mg/dl)*	0.34 [0.26, 0.36]	0.28 [0.24, 0.34]	0.20 [0.20, 0.24]^*^
*Urea (mg/dl)*	28 [22, 32]	27 [22, 32]	20 [18, 25]
*Sodium (mmol/l)*	137 [136, 140]	139 [138, 142]	139 [139, 142]
*Potasium (mmol/l)*	4.9 [4.6, 5.8]	5.3 [5.0, 5.5]	5.6 [5.1, 6.2]
**Arterial blood gases**			
*pH*	7.39 [7.33, 7.47]	7.44 [7.42, 7.48]	7.38 [7.33, 7,43]
*pCO*_*2*_ *(mmHg)*	46 [40, 51]	40 [36, 42]	51 [46, 57]^θθ^
*pO*_*2*_ *(mmHg)*	441 [341, 478]	455 [439, 478]	409 [336, 435]
*HCO*_*3*_ *(mmol/l)*	28.2 [27.0, 28.6]	27.3 [26.9, 27.8]	29.8 [27.8, 30.2]^θ^
*Lactate (mmol/l)*	1.0 [0.6, 1.2]	1.0 [0.6, 1.1]	0.9 [0.7, 1.0]

Values are Median [interquartile range]. Abbreviations: ALT, alanine transaminase; AP, alkaline phosphatase; AST, aspartate transaminase; HCO_3_, bicarbonate; INR, international normalized ratio; LDH, lactate dehydrogenase; MCV, mean corpuscular volume; pCO_2_, partial pressure of carbon dioxide; pO_2_, partial pressure of oxygen.

^#^1 rat in the CCl4-1xWk group and 2 in the CCl4-2xWk group had no laboratory data.

*p < 0.05 vs. Control, **p < 0.01 vs. Control, ***p < 0.001 vs. Control, ****p < 0.0001 vs. Control, ^θ^p < 0.05 vs. CCl4-1xWk, ^θθ^p < 0.01 vs. CCl4-1xWk, ^θθθθ^p < 0.0001 vs. CCl4-1xWk.

### Systemic hemodynamics

Both groups of rats receiving CCl4 presented stable systemic hemodynamics (heart rate, MAP and CVP) at the end of the experiments. The MAP, however, was higher in the CCl4-1xWk group compared with the Control (p < 0.05) and CCl4-2xWk (p < 0.01) groups (Table 3), suggesting that hyperdynamic circulation was more common in the CCl4-2xWk protocol. Rats in the CCl4-1xWk group showed mild increases of body temperature and respiratory rate (both p < 0.05 vs. Control) at the time of hemodynamic measurements, although all values were within the normal range (Table 3).

**Table 3:** General physiological and systemic hemodynamic parameters.

	Control	CCl4-1xWk	CCl4-2xWk
**General physiological parameters**			
Body temperature (°C)	36.6 [36.4, 37.3]	37.4 [37.1, 37.5]^**^	36.9 [36.6, 37.3]
Resp. rate (breaths per minute)	50 [46, 56]	58 [53, 59]^*^	46 [46, 60]
**Systemic hemodynamics**			
Heart rate (bpm)	318 [304, 363]	322 [293, 350]	303 [271, 333]
MAP (mmHg)	98 [83, 112]	112 [105, 118]^*^	92 [79, 95]^θ^
CVP (mmHg)	1.8 [0.9, 3.7]	2.2 [0.9, 2.9]	2.7 [0.6, 4.1]

Values represent the median [IQR]. ^*^p < 0.05 vs. Control, ^**^p < 0.01 vs. Control, ^θ^p < 0.05 vs. CCl4-1xWk. Abbreviations: bmp, beats per minute, CVP, central venous pressure, mmHg, millimeters of mercury, Resp. rate, respiratory rate.

### Development of fibrosis and cirrhosis of the liver

**Figure 3:**
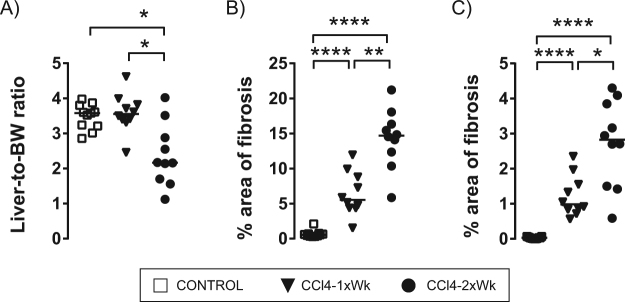
Changes in liver mass and development of fibrosis. The graphs show the liver-to-body weight ratio **(A)**, and the area of fibrosis (%) evaluated in liver tissue sections stained with **(B)** Sirius Red or **(C)** Masson’s Trichrome in rats receiving oral gavage with water (Control group), CCl4 once a week (CCl4-1xWk group) or CCl4 twice a week (CCl4-2xWk) for 12 weeks following the corresponding protocols. Horizontal lines represent the median. *p < 0.05, **p < 0.01, ****p < 0.0001 (ANOVA with Tukey’s post-hoc tests).

**Figure 4:**
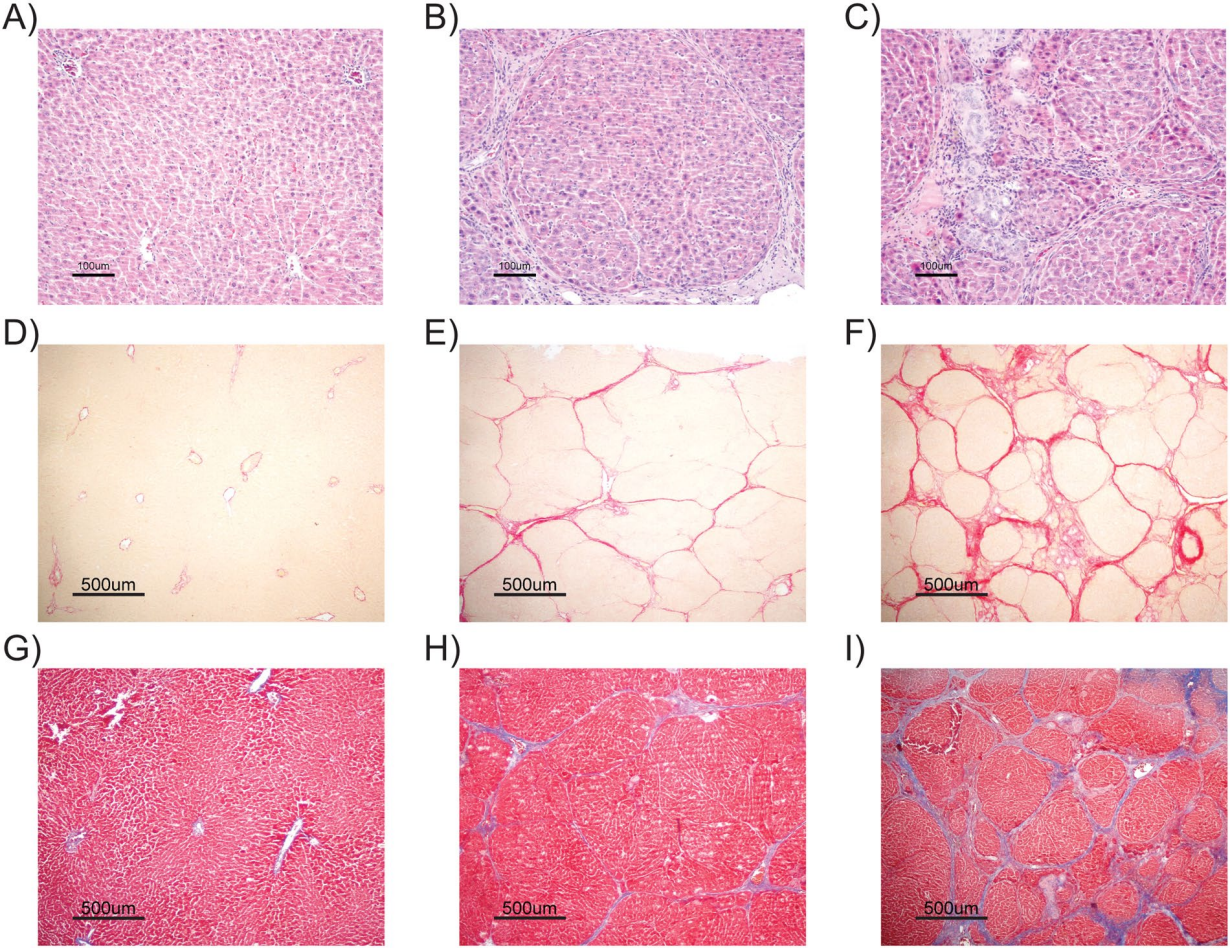
Representative histological pictures of liver tissue sections stained with (**A–C**) hematoxylin-eosin (H&E), (**D–F**) Sirius Red, and (**G–I**) Masson’s Trichrome. Liver samples were obtained at the termination of the experiments in rats that received (**A,D,G**) oral gavage with water (Control group), (**B,E,H**) CCl4 once a week (CCl4-1xWk group) or (**C,F,I**) CCl4 twice a week (CCl4-2xWk) for 12 weeks following the corresponding protocols. Lines in the lower-left corners represent 100 μmm (H&E staining, 10x objective) or 500 μm (Sirius Red and Masson’s Trichrome staining, 2x objective).

**Figure 5:**
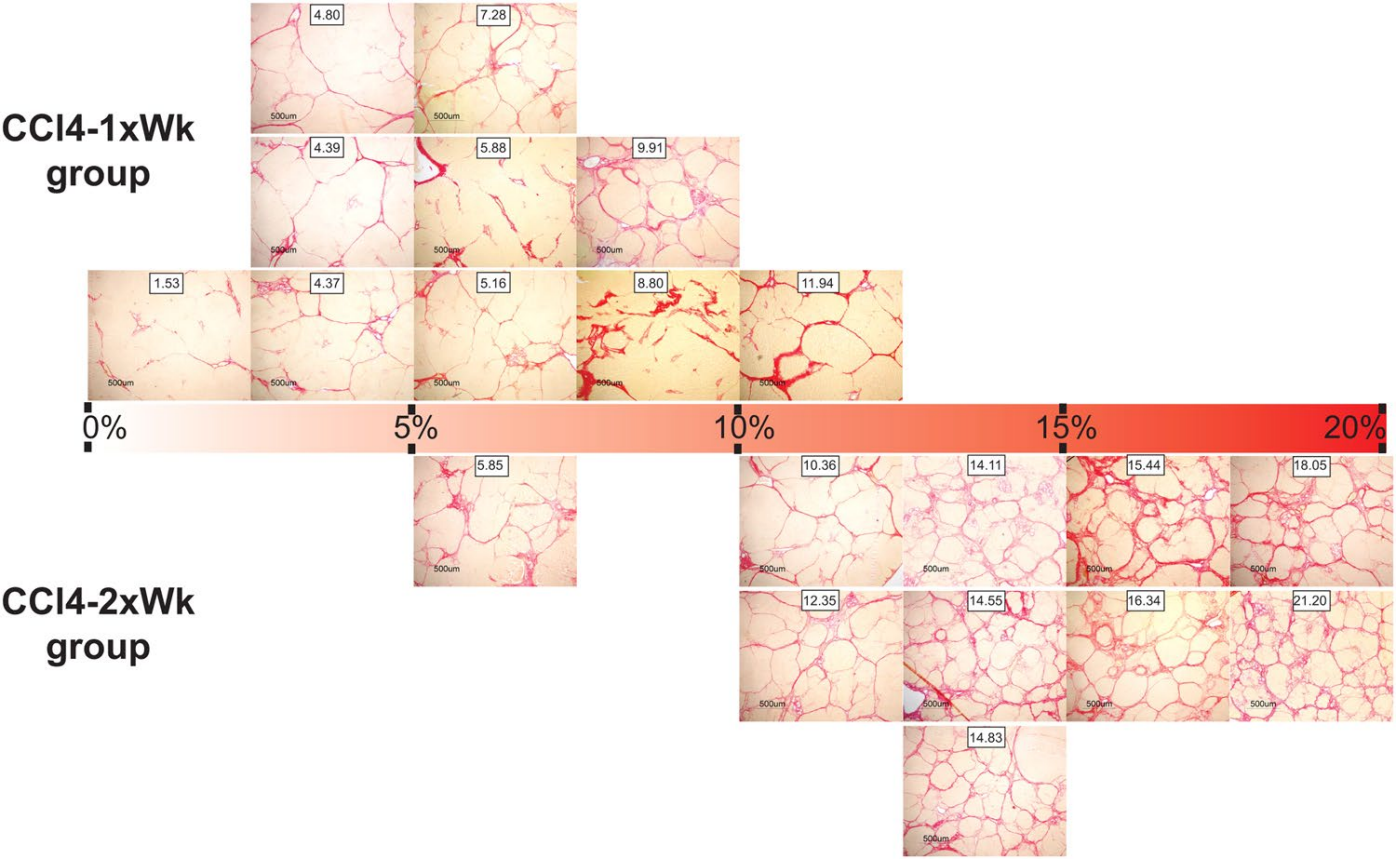
Schematic showing representative images of Sirius Red-stained liver sections from each rat receiving carbon tetrachloride. The rectangle with the red gradient in the middle represents the % area of hepatic fibrosis. Each image represents one rat, with their position being determined by the median area of fibrosis of the rat (shown in the inset). Rats following the CCl4-1xWk and the CCl4-2xWk protocols are shown, respectively, above and below the rectangle. Note that the extent of fibrosis in rats of the CCl4-1xWk group ranged from mild to severe, whereas the majority of rats in the CCl4-2xWk group presented severe fibrosis with fully developed cirrhosis in all of them. Within the CCl4-1xWk group, the rats showing 1.53% and 5.88% area of fibrosis did not fulfill histological criteria of cirrhosis.

Only the rats following the CCl4-2xWk protocol developed a marked atrophy of the liver, reflected by the lower liver-to-body weight ratios (2.17% [1.7, 3.0]) compared with those found in the Control (3.6% [3.2, 3.8]) and the CCl4-1xWk (3.6% [3.4, 3.9]) groups (both p < 0.05 vs. CCl4-2xWk, Fig. 3A). Most rats following the CCl4-1xWk protocol presented large nodules and thin fibrous septa, whereas all rats following the CCl4-2xWk protocol showed advanced macro-micronodular cirrhosis with remarkable architectural distortions and ductular proliferation (Fig. 4A–C). Both protocols of CCl4 administration induced hepatic fibrogenesis compared with Control rats, but the area of fibrosis was significantly higher in the CCl4-2xWk group than in the CCl4-1xWk group, both in liver sections stained with Sirius Red (CCl4-1xWk vs. CCl4-2xWk: 5.5% [4.4, 9.1] vs. 14.7% [11.9, 16.8], p < 0.01, Figs 3B and 4D–F) as well as in liver sections stained with Masson’s Trichrome (CCl4-1xWk vs. CCl4-2xWk: 0.99% [0.81, 1.66] vs. 2.83% [1.48, 3.91], p < 0.05, Figs 3C and 4G–I). A schematic with representative images of liver sections stained with Sirius Red from all rats showing the range of alterations in each group are shown in Fig. 5. Importantly, a histological diagnosis of cirrhosis was absent in two rats of the CCl4-1xWk group compared with the 100% yield of advanced cirrhosis in rats following the CCl4-2xWk protocol.

### Development of portal hypertension

**Figure 6:**
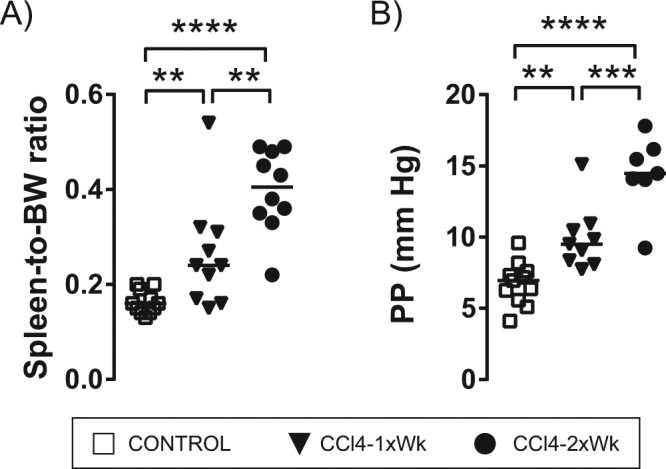
Development of splenomegaly and portal hypertension. The graphs show (**A**) changes of spleen mass evaluated by the spleen-to-body weight ratio, and (**B)** the portal pressure in rats receiving oral gavage with water (Control group), or CCl4 once (CCl4-1xWk group) or twice a week (CCl4-2xWk) for 12 weeks following the corresponding protocols. Horizontal lines represent the median of each group. **p < 0.01, ***p < 0.001, ****p < 0.0001 (ANOVA with Tukey’s post-hoc tests).

In line with the analytical and histological data, rats following the CCl4-2xWk protocol also showed increased portal pressure and portal hypertension-related complications such as ascites, splenomegaly and thrombocytopenia compared with rats in the CCl4-1xWk group. Thus, ascites was present in 0 of 11 (0%) Control rats, 1 of 10 (10%) CCl4-1xWk rats, and 8 of 10 (80%) CCl4-2xWk rats at the end of the experiments (p < 0.01 CCl4-2xWk vs. Control and CCl4-1xWk groups). Compared with Control rats, both groups of CCl4-1xWk and CCl4-2xWk rats developed splenomegaly (Fig. 6A) and portal hypertension (Fig. 6.B), but both parameters were higher in the rats following the CCl4-2xWk protocol (*Spleen-to-body weight ratio:* 0.16% [0.14, 0.19] vs. 0.24% [0.17, 0.31] vs. 0.41% [0.35, 0.48], p < 0.01 CCl4-1xWk vs. Control and CCl4-2xWk, p < 0.0001 CCl4-2xWk vs. Control; *Portal pressure:* 7.0 mmHg [5.6, 7.6] vs. 9.5 mmHg [8.2, 10.7] vs. 14.5 mmHg [14.0, 16.2], p < 0.01 CCl4-1xWk vs. Control, p < 0.001 CCl4-1xWk vs. CCl4-2xWk, p < 0.0001 CCl4-2xWk vs. Control).

## Discussion

Repeated exposition to CCl4 in the rat is a cardinal experimental animal model of cirrhosis, but its reproducibility problems and excessive mortality are well known^4,7^. In the present study, we performed a side-by-side comparison of one of the most commonly used CCl4 protocols involving oral gavage once a week (CCl4-1xWk)^6^ with a protocol involving oral gavage twice a week (CCl4-2xWk) based on the protocol reported by Regimbeau *et al*.^9^. The main finding of our study was that the CCl4-2xWk protocol was highly reproducible and largely superior to the CCl4-1xWk protocol in terms of development of advanced cirrhosis, portal hypertension and ascites at 12 weeks of treatment without worsening mortality.

Reproducibility is a cornerstone of experimental models that facilitates the accomplishment of solid conclusions. Despite its low reproducibility, the experimental model of CCl4-induced cirrhosis has provided relevant insight into mechanisms of liver disease because CCl4-treated rats behave very different than normal rats despite the presence of widely heterogenous degrees of liver fibrosis in the former. Such heterogeneity, however, becomes a serious problem when assessing the effect of different treatments or manipulations within CCl4-treated animals, a problem increased due to the limited sample size of most experimental studies. Importantly, two of the rats (20%) following the CCl4-1xWk protocol had liver fibrosis but did not develop cirrhosis in our study. Furthermore, different degrees of portal hypertension and histological (see schematic in Fig. 5) or biochemical alterations ranging from normal to markedly disturbed were observed within the CCl4-1xWk group, with only 20% of the rats presenting ascites after the 12-week period. A common approach to solve this issue and obtain homogeneous groups with cirrhosis consists of treating the rats with CCl4 until they develop overt ascites^14–18^, but this introduces additional problems. First, the total dose of CCl4 is highly different between the rats. Second, the different age and body weight of the rats when the experiments are performed influence many variables, as the duration of treatment may vary from 7 to more than 20 weeks. Third, the duration of concomitant treatments or manipulations will also largely differ within single groups. Fourth, the appearance of overt ascites is not always immediately detectable *de visu*, particularly in older rats. Finally, some rats following the CCl4-1xWk protocol never develop ascites or die earlier due to other complications representing a relevant bias^17^. In contrast to this scenario, all rats following the CCl4-2xWk protocol in our study developed advanced cirrhosis and portal hypertension within 12 weeks of CCl4 administration, with ascites being confirmed in 80% of them. This was further supported by the presence of liver atrophy and the characteristic alterations of cirrhosis in blood analyses, including parameters related to liver damage (transaminases, AP), liver function (bilirubin, INR, albumin) or portal hypertension and hypersplenism (thrombocytopenia).

Despite the accelerated development of advanced cirrhosis in rats following the CCl4-2xWk protocol, mortality was similar in both protocols, and compared favorably with mortality rates (30% to 60%) reported in most studies^6,14–18^. Notably, mortality may be further reduced as accidental instillation of CCl4 into the trachea, which occurred at a rate higher than expected according to our prior experience, was the cause of 3 of the 5 deaths (60%) in the CCl4-2xWk group. The smoother curve of body weight gain in the CCl4-2xWk group versus the serrated curve observed in the CCl4-1xWk group also suggests that the lower and more repeated doses used in the former protocol mimicked better the development of a chronic disease versus the higher acute doses used in the CCl4-1xWk protocol.

It is important to note that not all experimental animal models of liver disease express the disturbances characteristic of the syndrome of portal hypertension or can be used in several species^4^. In this regard, the administration of CCl4 accomplishes most requirements, being a feasible and not expensive model with minimal extrahepatic damage that can be used in different species (rats, mice, rabbits) and that closely resembles the biochemical, histological and hemodynamic alterations observed in human patients^19^. Among the different routes of administration of CCl4, the orogastric route is frequently used as it presents several advantages versus other alternatives. In particular, the administration of CCl4 by oral gavage requires low quantities of CCl4 and ensures its direct delivery to the liver through the portal vein, diminishing the extrahepatic effects due to the selective accumulation of CCl4 in the liver^20^. The inhalational route is also frequently used^21,22^, but it exposes extra-hepatic organs to CCl4 and is more expensive due to the need of large amounts of CCl4 and specific facilities to perform the administration (fume-hoods, filters). Importantly, CCl4 causes liver toxicity in humans and was *reasonably anticipated* to be a human carcinogen in the 12^th^ Report on Carcinogens^23^. Finally, the intraperitoneal route is convenient, but it interferes hemodynamic measurements due to the formation of adhesions and has the risk of vessel puncture and intraabdominal bleeding particularly in advanced liver disease presenting large portal-systemic collaterals.

In conclusion, our results are important for liver research as they validate the utility of a modified version of the protocol described by Regimbeau *et al*.^9^ compared to a more widely used but inferior protocol described by Runyon *et al*.^6^. In particular, the CCl4-2xWk protocol accomplished the rapid development of advanced cirrhosis, portal hypertension and ascites in rats over a 12-week period of exposition to CCl4 in a highly effective and reproducible manner, without worsening mortality.

## References

[1] Iredale JP (2007). Models of liver fibrosis: exploring the dynamic nature of inflammation and repair in a solid organ. J. Clin. Invest..

[2] Vorobioff J, Bredfeldt JE, Groszmann RJ (1983). Hyperdynamic circulation in portal-hypertensive rat model: a primary factor for maintenance of chronic portal hypertension. Am. J. Physiol..

[3] Okazaki I, Maruyama K (1974). Collagenase activity in experimental hepatic fibrosis. Nature.

[4] Abraldes J-G, Pasarín M, García-Pagán J-C (2006). Animal models of portal hypertension. World J. Gastroenterol..

[5] Liu Y (2013). Animal models of chronic liver diseases. Am. J. Physiol. Gastrointest. Liver Physiol..

[6] Runyon BA, Sugano S, Kanel G, Mellencamp MA (1991). A rodent model of cirrhosis, ascites, and bacterial peritonitis. Gastroenterology.

[7] Proctor E, Chatamra K (1982). High yield micronodular cirrhosis in the rat. Gastroenterology.

[8] Chatamra K, Proctor E (1981). Phenobarbitone-induced enlargement of the liver in the rat: its relationship to carbon tetrachloride-induced cirrhosis. Br. J. Exp. Pathol..

[9] Regimbeau J-M, Fuks D, Kohneh-Shahri N, Terris B, Soubrane O (2008). Restrictive model of compensated carbon tetrachloride-induced cirrhosis in rats. World J. Gastroenterol..

[10] Desmyter L (2007). Rating of CCl(4)-induced rat liver fibrosis by blood serum glycomics. J. Gastroenterol. Hepatol..

[11] Masuda H, Fukumoto M, Hirayoshi K, Nagata K (1994). Coexpression of the collagen-binding stress protein HSP47 gene and the alpha 1(I) and alpha 1(III) collagen genes in carbon tetrachloride-induced rat liver fibrosis. J. Clin. Invest..

[12] Sogni P (1998). Beneficial hemodynamic effects of bosentan, a mixed ET(A) and ET(B) receptor antagonist, in portal hypertensive rats. Hepatol. Baltim. Md.

[13] Wang YJ (1998). Two novel antifibrotics, HOE 077 and Safironil, modulate stellate cell activation in rat liver injury: differential effects in males and females. Am. J. Pathol..

[14] Bartolí R (2007). Effect of the administration of fermentable and non-fermentable dietary fibre on intestinal bacterial translocation in ascitic cirrhotic rats. Clin. Nutr. Edinb. Scotl..

[15] Guarner C, Runyon BA, Heck M, Young S, Sheikh MY (1999). Effect of long-term trimethoprim-sulfamethoxazole prophylaxis on ascites formation, bacterial translocation, spontaneous bacterial peritonitis, and survival in cirrhotic rats. Dig. Dis. Sci..

[16] Llovet JM (1994). Bacterial translocation in cirrhotic rats. Its role in the development of spontaneous bacterial peritonitis. Gut.

[17] Pérez-Paramo M (2000). Effect of propranolol on the factors promoting bacterial translocation in cirrhotic rats with ascites. Hepatol. Baltim. Md.

[18] Úbeda M (2010). Critical role of the liver in the induction of systemic inflammation in rats with preascitic cirrhosis. Hepatol. Baltim. Md.

[19] Constandinou C, Henderson N, Iredale JP (2005). Modeling liver fibrosis in rodents. Methods Mol. Med..

[20] Recknagel RO, Litteria M (1960). Biochemical changes in carbon tetrachloride fatty liver: concentration of carbon tetrachloride in liver and blood. Am. J. Pathol..

[21] Fortea JI (2018). Enoxaparin does not ameliorate liver fibrosis or portal hypertension in rats with advanced cirrhosis. Liver Int. Off. J. Int. Assoc. Study Liver.

[22] Tripathi DM (2015). Metformin reduces hepatic resistance and portal pressure in cirrhotic rats. Am. J. Physiol. Gastrointest. Liver Physiol..

[23] National Toxicology Program. NTP 12th Report on Carcinogens. *Rep. Carcinog. Carcinog. Profile*s **12**, iii-499 (2011). 21822324

